# A randomised controlled trial to explore attitudes to routine scale and polish and compare manual versus ultrasonic scaling in the general dental service in Scotland [ISRCTN99609795]

**DOI:** 10.1186/1472-6831-5-3

**Published:** 2005-06-23

**Authors:** Brian C Bonner, Linda Young, Patricia A Smith, Wendy McCombes, Jan E Clarkson

**Affiliations:** 1 The Dental Health Services Research Unit, Mackenzie Building, Kirsty Semple Way, Dundee DD2 4BF, UK; 2 Postgraduate Dental Office, Eastern Region Dental Postgraduate Centre, Dundee Dental Hospital and School, Park Place, Dundee DD1 4HN, UK; 3 Scottish Dental Practice Based Research Network, The Lister Postgraduate Institute, Edinburgh EH8 9DR, UK

## Abstract

**Background:**

To investigate, within general dental practice, patients' and vocational dental practitioners' (VDP) attitudes towards the benefits and costs of a simple scale and polish and to compare the experience of using manual versus ultrasonic instruments to scale teeth.

**Methods:**

28 VDPs and 420 patients participated. Patients were randomly allocated to either group. Patients' and VDPs' attitudes towards, and experience of, the scale and polish were elicited by means of self-administered questionnaires.

**Results:**

The majority of patients (99%) believed a scale and polish was beneficial. VDPs considered ultrasonic treatment to be appropriate on significantly more occasions than they did for manual scale and polish (P < 0.001). Patient discomfort: with ultrasonic scaling 69.2% felt 'a little uncomfortable' or worse compared with 60% of those undergoing manual treatment (P = 0.072). VDPs considered treatment charges were appropriate for 77% of patients.

**Conclusion:**

Routine scaling and polishing is considered beneficial by both patients and vocational trainees. The majority of patients, regardless of treatment method, experience some degree of discomfort when undergoing a scale and polish. VDPs showed a preference for the ultrasonic treatment method.

## Background

The Scottish Dental Practice Based Research Network's (SDPBRN) Vocational Dental Practitioner (VDP) Practice Based Research Programme is a new initiative in which VDPs are invited to take part in practice-based research studies [1]. A key aim of the programme is to encourage the development of an interest in the link between improvements in primary dental care and the findings of good quality practice-based research. The randomised controlled trial (RCT) reported here, which was carried out in the North and North-East of Scotland, was the first study in this programme. As such, it was a pilot trial: the programme having subsequently been extended Scotland-wide.

The need for practice-based research in primary dental care is widely recognised, but significant barriers do exist [2]. One such is the perceived difficulty of conducting studies, such as RCTs, without disrupting clinical work and patient care, and this can lead to a general reluctance to become involved [3]. Despite this reservation, if the evidence base in dental primary care is to be improved and if general dental practitioners (GDPs) are to be able to assess the generalisability of research results to their own practice, then high quality practice-based RCTs must be conducted within primary dental care.

During the year ending March 2002, GDPs within the General Dental Service in Scotland carried out 1,376,500 simple scale and polishes (Item 10a; Statement of Dental Remuneration) at a total cost, to health boards and patients, of £13,874,109. In contrast, only 107,042 scales and polishes requiring a minimum of two visits (Item 10b) and 2,037 intensive scales including periodontal charting (Item 10c) were provided. This typifies one aspect of the existing long-term pattern of primary care treatment, where the majority of periodontal treatment currently carried out in general dental practice consists solely of simple scaling and polishing [4].

In the 1988 UK Adult Dental Health Survey, 60% of Scottish Adults (72%; UK) were found to have visible plaque on their teeth, 62% calculus (73%; UK), and 47% periodontal pocketing of 4 mm or more (54%; UK) [5]. A lower proportion of those individuals who had visited a GDP regularly had visible plaque and calculus but this was also the case with people who reported good, self-administered dental hygiene. In recent years, the clinical need for the large numbers of simple scales and polishes carried out in the GDS has been questioned. The 2002 Audit Commission Report on the primary dental care services in England and Wales [6] suggests that in excess of half the simple scale and polish treatments prescribed may be unnecessary and may not lead to any health gain.

Prior research has concentrated on the effects of scale and polish on periodontal health [7-11]. Little research has been carried out into the attitudes of patients or GDPs towards this treatment. This trial was designed to address this gap in the knowledge base by investigating, within general dental practice vocational training GDPVT), both patients' and VDPs' attitudes towards routine scale and polish, and by comparing the experience, again from both patients' and VDPs' viewpoints, of using either manual or ultrasonic techniques.

## Methods

The trial protocol was developed in collaboration with the regional dental vocational training (DVT) adviser for the East and North East of Scotland and the GDPVT adviser for each participating DVT scheme. A key consideration, when developing the protocol, was to limit disruption of the normal routine of the surgery as much as possible. Therefore, a pragmatic approach was taken, with one potential advantage of this approach being the generalisability of the results to 'real world' general dental practice.

### Participants

The trial was conducted from April to June 2001. All 28 VDPs in the Aberdeen, Dundee, and Perth DVT schemes were invited to participate. Each VDP was asked to recruit 16 patients. All adult patients who were dentate, generally fit and well, attending for a routine check-up appointment, and who, in the VDP's clinical opinion, required a simple scale and polish were eligible for inclusion in the study. This treatment is defined as: 'non-surgical treatment involving scaling, polishing, and simple periodontal treatment including oral hygiene instruction, requiring only one visit'. A patient's eligibility was determined only after examination by the VDP. If the patient's proposed treatment plan did not include a simple scale and polish, the patient was not invited to participate. No attempt was made to influence this decision.

### Randomisation

Consenting patients were allocated to either the manual or ultrasonic scaling group. To improve the balance of the trial arms a computer generated block randomisation sequence was used. In this, scaling allocations were generated in groups of eight with four participants allocated, in a random sequence, to each intervention group. Scaling group allocation was concealed in an opaque envelope, which was not opened until the patient agreed to take part. At the behest of either the patient or the clinician, the patient could be transferred to the other treatment method and any such changes were noted.

### Materials and interventions

All patients invited to participate were encouraged to read a laminated patient information sheet, accompanied by a verbal explanation of the study. Patient characteristics, age, sex, and number of teeth, were recorded on the patient recruitment form. When patients declined to take part, the reason was noted.

Individual patient and VDP trial questionnaires, consisting chiefly of closed single or multiple response questions, were developed following a review of the literature and in collaboration with the DVT regional and scheme advisers. A pilot study was carried out with the help of a number of GDPs and their patients and several small changes were made to both questionnaires before the trial commenced.

Questionnaires were self-administered and investigated reasons for carrying out the scale and polish and attitudes towards this treatment from both the patient and VDP viewpoints.

Both parties filled out questionnaires once the treatment had been completed and then concealed them in opaque envelopes.

### Power calculation

When deciding how many subjects to include in each arm of the trial, a pragmatic approach was adopted, as no information was available from previous studies to indicate the proportions of patients who would select each option for the questions they were to be asked. Advice from regional and GPVT advisers indicated that it was reasonable for each VDP to recruit 16 patients, totalling 448 patient participants. This number of subjects is sufficient to detect differences of approximately 15% between proportions with a power of 80% (α = 0.05) [12].

### Ethical approval

The authors were advised, by the Chairperson of the Local Ethics Committee, that as this was a study of routine treatment that had been prescribed for the patient solely on the grounds of clinical need, with positive consent for inclusion in the study of all participants, formal ethical approval was not required.

## Results

### Participant flow

In total, 420 patients (out of 442 invited) agreed to participate (Male n = 178, mean age 41, range 17–76; Female n = 240, mean age 38, range 16–80; both male and female had a mean number of 24 teeth, range 5–32; missing = 2). Reasons for refusal were varied and included insufficient time and no interest in participating, with one patient concerned that ultrasonic scaling would damage his restorations. Nineteen VDPs recruited 16 patients, four recruited 15 patients, two recruited 13 patients, two recruited 12 patients, and one recruited 6 patients.

**Figure 1:**
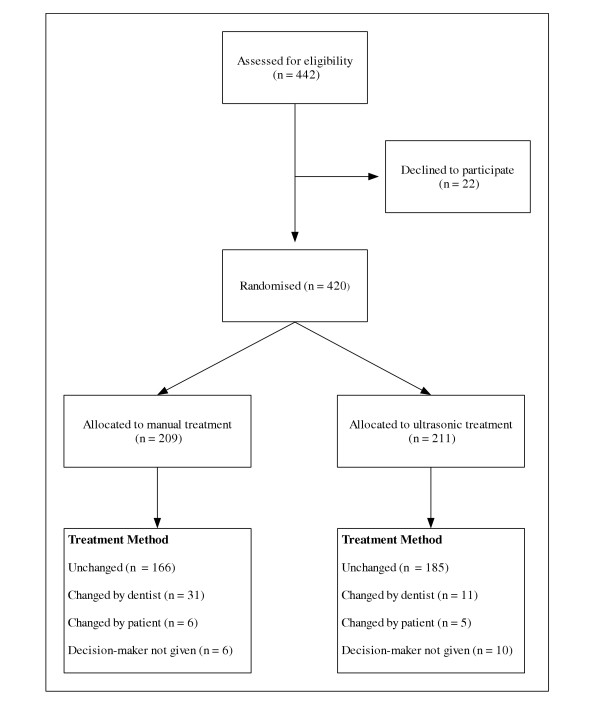
Flow of patients through the randomised controlled trial.

Figure 1 summarises the flow of participants through the study. Two hundred and nine patients were allocated to the manual group and 211 patients to the ultrasonic instrument group. The scaling method was changed from one group to the other on 69 occasions. The number of patients who crossed over from one arm of the trial to the other was higher than anticipated, leading to the formation of a third group, named the crossover group (n = 69). These patients were excluded where comparisons were made between the manual (n = 166) and ultrasonic (n = 185) treatment, but included where appropriate.

### Benefits of a routine scale and polish

Patients were offered six options to elicit their reasons for having a scale and polish (multiple responses allowed). In descending order the response to, "Why did you have a scale and polish?" were tartar 191 patients (46%), stained teeth 150 patients (36%), bleeding gums 82 patients (20%), mouth felt unclean 65 patients (16%), and gum disease 55 patients (13%). An alternative response, "always have one with a check up" was selected by 183 patients (44%). For 91 (22%) patients, this was the only reason chosen.

**Figure 2:**
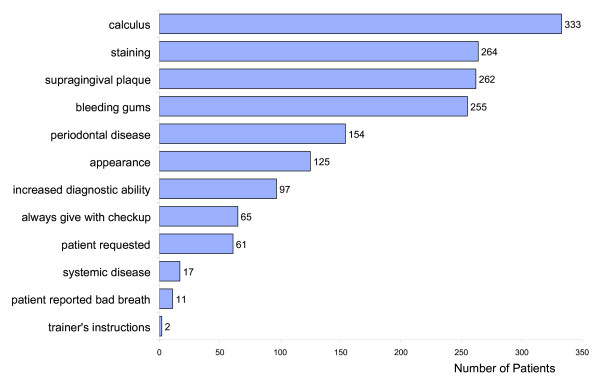
"Why did you provide a scale and polish for this patient?"

Figure 2 shows, for each patient, the reasons why the VDP prescribed the scale and polish. The predominant reason given was that calculus was present 333 patients (79%). Other reasons included staining 264 patients (63%), supragingival plaque 262 patients (62%), bleeding gums 255 patients (61%), periodontal disease 154 patients (37%), appearance 125 patients (30%), and to increase diagnostic ability 97 patients (23%).

Questions concerning patient's perceptions of how their teeth looked and felt following the scale and polish revealed that there were no significant differences between the manual and ultrasonic groups in their responses (table 1).

**Table 1:** Showing patients perception of how their teeth looked and felt after they had received a scale and polish.

	Manual % (n = 166)	Ultrasonic % (n = 185)		P	Crossover (%) (n = 69)
Feel cleaner	88	89	0.001	0.971	87
Feel smother	72	65	1.62	0.203	73
Feel the same	2	3	*	0.727	3
Feel worse	0	2	*	0.250	3
Look better	49	55	0.75	0.386	61
Look the same	4	7	0.493	0.483	4
Look worse	0.006	0.005	*	> 0.999	0

*: Fisher's Exact Test

**Figure 3:**
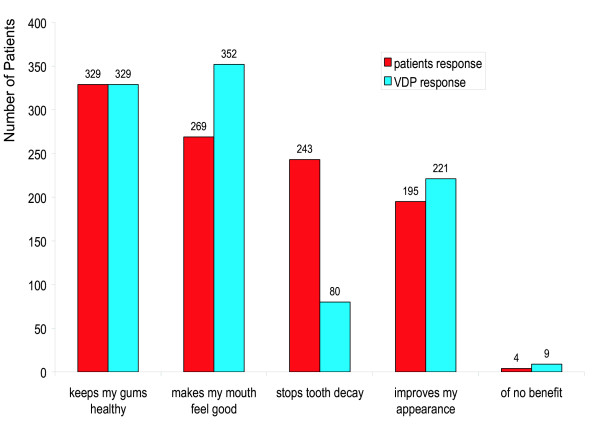
Perceived benefit of scale and polish. Patients: "What do you think is the benefit to you of having a scale and polish?" Dental vocational practitioners: "How do you think the patient will feel they benefited from this scale and polish?"

Related closed multiple response questions, each with the same five possible response options, were posed to the patient and VDP regarding the patient's perception of the benefits of a scale and polish. The patient was asked "What do you think is the benefit to you of having a scale and polish?" and, to elicit the VDP's appreciation of the patient's perceptions, the VDP was asked "What do you think the patient will feel they benefited from having this scale and polish?". Four patients (1%) believed that a scale and polish was 'of no benefit' to them, 195 patients (46%) believed a scale and polish 'improves appearance', and 329 patients (78%) believed a scale and polish would keep their 'gums healthy'. Whilst anticipating these responses, VDPs did not expect that as many as 243 patients (58%) would believe that scaling and polishing was instrumental in arresting tooth decay, and over-estimated the number of patients who felt it made their mouths 'feel good' (Figure 3).

### Costs of a routine scale and polish

**Figure 4:**
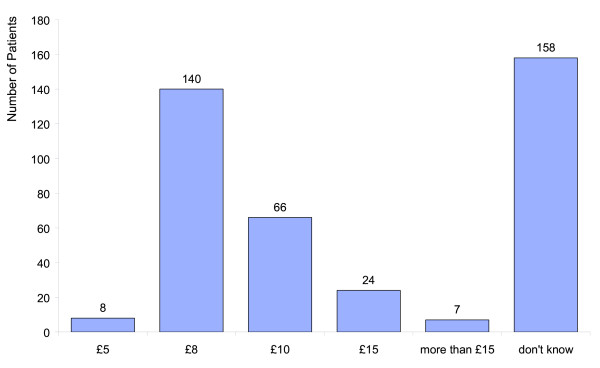
Patient question: "How much did the scale and polish cost?"

The patient's responses to the question "How much did the scale and polish cost? (from a choice of £5, £8, £10, £15, or more)" indicates that the majority of patients were unaware of the price paid for individual items of treatment. While 33% of patients correctly answered that a scale and polish cost £8, 25% gave an incorrect response and 38% answered 'don't know'. The remaining four percent declined to answer (Figure 4).

Patients were also asked "How much would you be prepared to pay for a scale and polish?" (from a choice of £5, £8, £10, £15, or more). Those receiving a manual scale indicated a willingness to pay slightly more and this difference became more apparent on comparing the proportion of each group who were prepared to pay £10 or more: 69% (109/158) from the manual group compared with 59% (101/170) from the ultrasonic group, though the difference was not statistically significant (, 2.857, P = 0.091). Twenty three patients (manual n = 8; ultrasonic n = 15) did not respond to this question.

VDP's considered that the patient charge for treatment was appropriate for 82% (132/161) of manual treatments and for 78% (142/182) of ultrasonic treatments ( = 0.836, P = 0.361; appropriate for 77% overall when the cross-over group was included). When the charge was considered inappropriate, the suggested alternative charge was higher than £8 in 90% of cases, regardless of treatment group.

### Experience of manual versus ultrasonic treatment

VDPs considered that the allocated method of scaling was the most appropriate for a significantly higher proportion of the ultrasonic group (189/210) than for the manual group (111/166;  = 40.3, P < 0.001).

Crossover was significantly less from the ultrasonic to the manual group (26/202) than vice versa (43/202), ( = 4.47, P = 0.03). VDPs gave a variety of reasons for their decision to alter the scaling method. Generally, increased speed and efficiency of stain removal was used to justify change to ultrasonic scaling and tight contact points and pain were the principal reasons for crossover from ultrasonic to manual scaling. When the change was patient requested, the reason given, regardless of the direction of crossover, was that the allocated scaling method was painful.

Patients were asked "What did the scale and polish feel like?" (choosing from comfortable, a little uncomfortable, very uncomfortable, and painful). In general, patients felt greater discomfort with ultrasonic scaling with 69% (126/182) of patients feeling 'a little uncomfortable' or worse compared with 60% (99/165) of those undergoing manual scaling, but this was not statistically significant ( = 3.24, P = 0.072).

## Discussion

This pilot trial was designed to compare the attitudes of VDPs and their patients towards two alternative, randomly allocated, simple scale and polish procedures provided at general dental practices in Scotland, under normal day-to-day conditions.

For pragmatic reasons, the patients included in this study were not stratified in any way and no weighting to reflect proportions of local populations examined was considered. Nevertheless, the patients recruited to the trial were seen to be comparable, in general characteristics, with patients attending for National Health Service (NHS) dental treatment in Scotland with an average of 24 teeth (24; Adult Dental Health Survey, 1998) [5], mean age 40 years (41 Adult Dental Health Survey, 1998) [5], and male to female ratio of 1:1.34 (1:1.36; General Household Survey, 1991) [13].

The VDPs gave a variety of reasons why they thought it necessary for their patients to receive a scale and polish chief amongst which were calculus, staining, supragingival plaque, and bleeding gums. In contrast to the Audit Commission Report suggesting that more than half of the simple scales and polishes carried out in the GDS are unnecessary and confer no health benefits [6], the patients surveyed in this trial had all been judged by their clinician to require a scale and polish. The patients, including the 91 patients whose only reason for having a scale and polish was because they "always have one with a check up", also generally believed that they would benefit from a scale and polish, with only 1% of patients believing a scale and polish was of no benefit. The evidence supporting the benefit of frequent scale and polish has, however, been questioned [14]. A high proportion of patients (58%) expected the scaling and polishing to stop tooth decay: an opinion not always appreciated by their dentist as only in one fifth of cases were patients predicted to give this response.

VDPs appeared to show a preference for the ultrasonic treatment and were more likely to transfer the patient from hand to ultrasonic treatment than vice versa. They suggested that the ultrasonic treatment was more efficient in terms of speed of treatment and more effective where contact points were tight. A recent review of a number of, mostly hospital-based, comparisons between these two techniques did note a moderate time saving [15]. The comfort of patients during scaling should be considered, as many nervous dental patients apparently find dental hygiene treatment contributes greatly to their anxiety towards visits for dental treatment [16]. The spread of the patients' experience between comfortable and in pain was similar for the two treatments and similar small numbers asked to be transferred to the other treatment because of pain.

In the UK, dental care is provide either privately or within the NHS at a fixed cost that includes a patient contribution of 80% (up to a maximum charge for a course of treatment). Around 49% of the population are registered with an NHS dentist at any one time [4] but, as registration lapses if a visit is not made within 15 months, a greater proportion of the population is believed to receive care within the NHS dental service than this figure might suggest. Although the number of dental treatments provided outside the NHS scheme is increasing in the UK in general, in Scotland an estimated 81% of treatments are currently provided under the NHS system [5] and it is likely that the majority of the population will continue to seek NHS dental care. All the patient participants in this study were receiving a scale and polish under the NHS system, for which a charge of £8.08 was in place at the time of the study. Although the majority of patients were unaware of the price paid for individual items of service, many patients did seem to be aware of the approximate cost of their treatment, with 49% of the subjects suggesting either £8 or £10 as the cost of a scale and polish. Slightly more subjects in the manual treatment group said they would be prepared to pay more than in the ultrasonic group possibly indicating a feeling that this treatment involved more activity by the VDP and was therefore 'worth' more.

Undoubtedly, there are difficulties in conducting studies in general dental practice, including time pressures to both patients and dentists and the need to fit in with the priority of providing good patient care. However, this pilot trial has shown that primary care-focused studies can be successfully carried out and VDPs taking part ended up with a more positive view of the concept of undertaking research in the dental surgery (findings of focus groups: not detailed here). Useful information has emerged on the attitudes and beliefs of GDS patients and newly qualified dentists towards simple scaling and polishing and the co-ordinators of the SDPBRN VDP Practice Based Research Programme have been sufficiently encouraged to commit to further studies in this series on a Scotland-wide basis.

## Conclusion

The results have demonstrated that routine scaling and polishing is considered to be beneficial by both patients and VDPs and that the majority of patients, regardless of whether they received ultrasonic or manual treatment, experience some degree of discomfort. VDPs showed a preference for the ultrasonic treatment method.

This study has also demonstrated that it is possible, with careful choice of research topic and a pragmatic approach, to carry out meaningful research in a primary care setting.

## Competing interests

The author(s) declare that they have no completing interests.

## Authors' contributions

BCB and LY managed the day-to-day running of the study, production and distribution of questionnaires, and analysis of results; PAS was responsible for suggestions towards questionnaires and conduction of de-briefing interviews with VDPs; WM is the regional training co-coordinator and liaised with and recruited VDPs to the study; and JEC was the principle investigator with overall responsibility for the study. All authors read and approved the final manuscript.

## Pre-publication history

The pre-publication history for this paper can be accessed here:


